# How to test for a relative afferent pupillary defect (RAPD)

**Published:** 2012

**Authors:** David C Broadway

**Affiliations:** Consultant ophthalmic surgeon, Department of Ophthalmology, Norfolk & Norwich University Hospital, and Honorary Reader, University of East Anglia, Norwich, UK.

**Figure:**
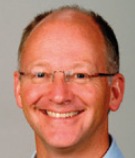
David C Broadway

The ‘swinging light test’ is used to detect a relative afferent pupil defect (RAPD): a means of detecting differences between the two eyes in how they respond to a light shone in one eye at a time. The test can be very useful for detecting unilateral or asymmetrical disease of the retina or optic nerve (but only optic nerve disease that occurs in front of the optic chiasm).

The physiological basis of the RAPD test is that, in healthy eyes, the reaction of the pupils in the right and left eyes are linked. In other words, a bright light shone into **one** eye leads to an equal constriction of **both** pupils. When the light source is taken away, the pupils of both eyes enlarge equally. This is called the **consensual light reflex.**

To understand how the pupils react to light, it is important to understand the **light reflex pathway** (Figure 1). This pathway has two parts.

1. The **afferent** part of the pathway (red) refers to the nerve impulse/message sent from the pupil to the brain along the optic nerve when a light is shone in that eye.
2. The **efferent** part of the pathway (blue) is the impulse/message that is sent from the mid-brain back to **both pupils** via the ciliary ganglion and the third cranial nerve (the oculomotor nerve), causing both pupils to constrict, even even though only one eye is being stimulated by the light.

**Figure 1:**
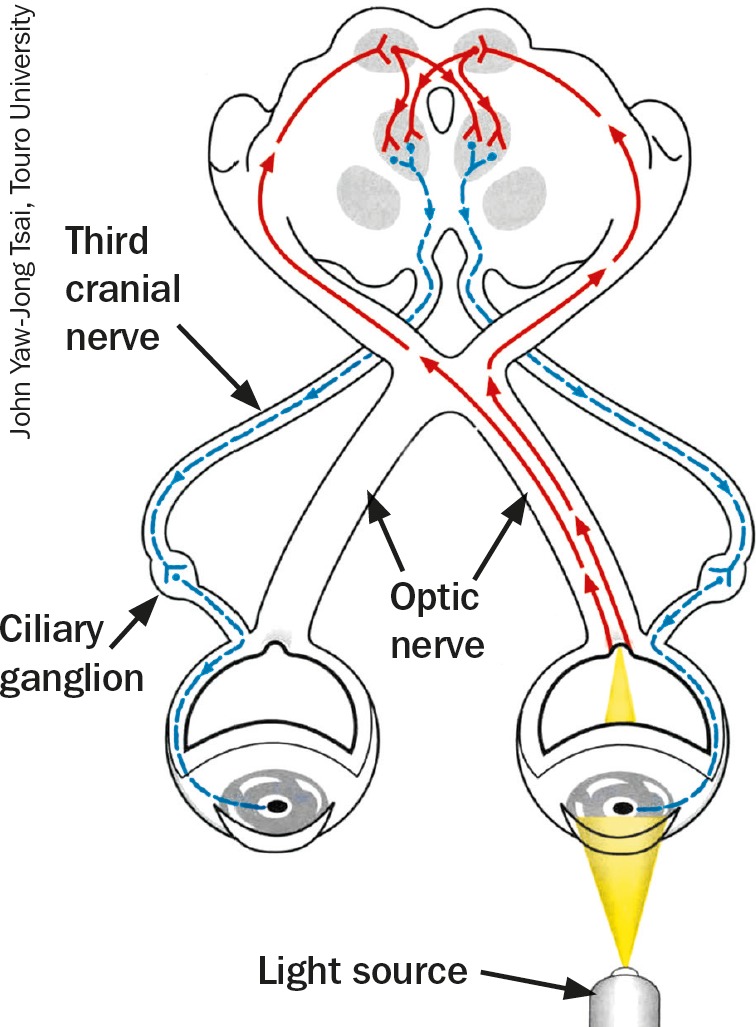
The light reflex pathway showing the afferent path (red) and the efferect path (blue)

A **positive** RAPD means there are differences between the two eyes in the afferent pathway due to retinal or optic nerve disease. If the light used is sufficiently bright, even a dense cataract or corneal scar will not give a RAPD as long as the retina and optic nerve are healthy. Indeed, the test can be used to assess the health of the retina and optic nerve behind a dense cataract, for example.

In glaucoma, if other tests of visual function (e.g. visual fields) are not possible, detecting a RAPD can be very useful as it indicates that there is more optic nerve damage in one eye than in the other, even if the visual acuity in both eyes is equal.

**NOTE: If the glaucomatous damage is equal in the two eyes, there will be no RAPD, however severe the damage is.**

## The swinging light test

**Figure 2:**
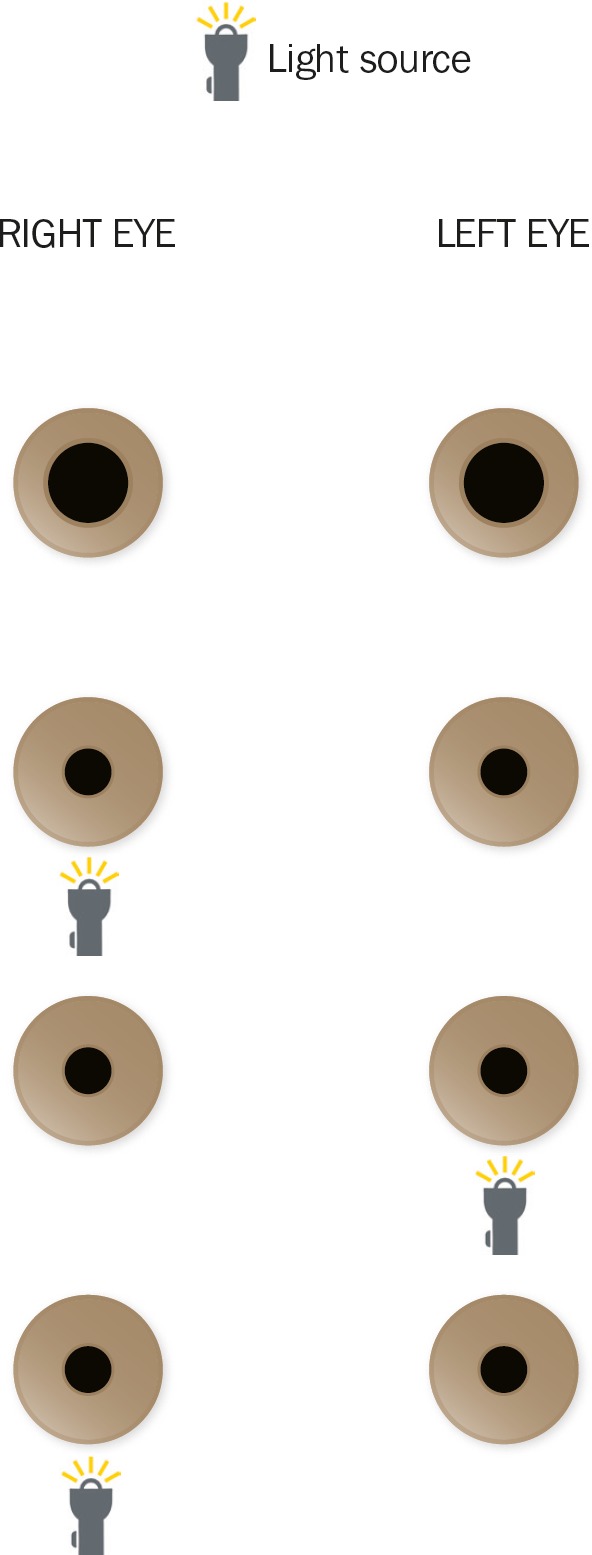
**Swinging-light test - normal (no RAPD)** Illumination of either eye induces normal and equal pupil responses in both eyes (consensual responses).

**Figure 3:**
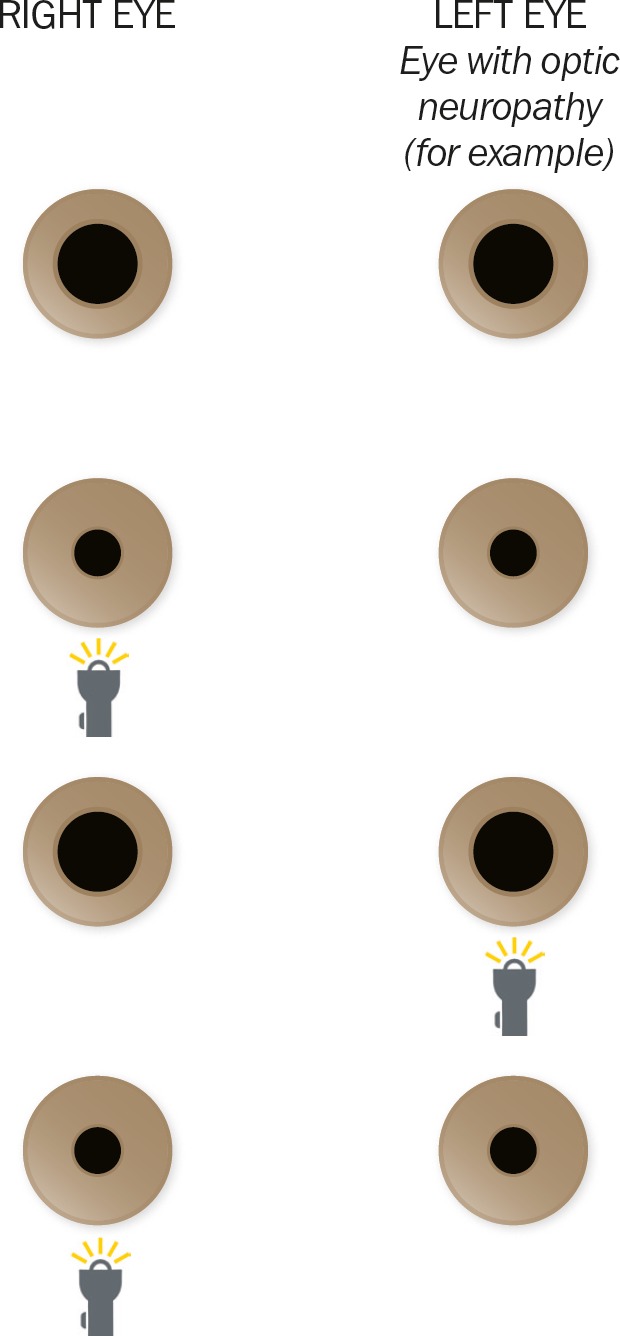
**Swinging-light test – left RAPD** Illumination of the (more) normal right eye causes both pupils to constrict. When the light is moved to the (more) abnormal left eye (e.g. with optic neuropathy), both pupils dilate (constrict less), the left pupil dilating despite the light being shone directly at it. Returning the light to the (relatively) normal right eye results in constriction of both pupils again.

In a normal swinging light test (i.e. there is no RAPD) the pupils of both eyes constrict equally regardless of which eye is stimulated by the light (Figure 2). In an abnormal swinging-light test (i.e. there is a RAPD) there is less pupil constriction in the eye with the retinal or optic nerve disease (Figure 3).

## Steps

- Use a bright torch which can be focussed to give a narrow, even beam of light. Perform the test in a semi-darkened room. If the room is too dark it will be difficult to observe the pupil responses, particularly in heavily pigmented eyes.
- Ask the patient to look at a distant object, and to keep looking at it. Use a Snellen chart, or a picture. This is to prevent the near-pupil response (a constriction in pupil size when moving focus from a distant to a near object). While performing the test, take care not to get in the way of the fixation target.
- **Move** the whole torch deliberately from side to side so that the beam of light is directed directly into each eye. **Do not swing** the beam from side to side around a central axis (e.g. by holding it in front of the person's nose) as this can also stimulate the near response.
- Keep the light source at the same distance from each eye to ensure that the light stimulus is equally bright in both.
- Keep the beam of light steadily on the first eye for at least 3 seconds. This allows the pupil size to stabilise. Note whether the pupil of the eye being illuminated reacts briskly and constricts fully to the light. Also note what happens to the pupil of the other eye: does it also constrict briskly?
- Move the light quickly to shine in the other eye. Again, hold the light steady for 3 seconds. Note whether the pupil being illuminated stays the same size, or whether it gets bigger. Note also what happens to the other eye.
- As there is a lot to look at, repeat the test, observing what happens to the pupils of both eyes when one and then the other eye is illuminated.

When the test is performed on someone with unilateral or asymmetrical retinal or optic nerve disease, a RAPD should be present (Figure 3). The following happens:

- When the light is shone into the eye with the retinal or optic nerve disease, the pupils of both eyes will constrict, but not fully. This is because of a problem with the afferent pathway.
- When the light is shone into the other, normal (less abnormal) eye, both pupils will constrict further. This is because the afferent pathway of this eye is intact, or less damaged than that of the other eye.
- When the light is shone back into the abnormal eye, both pupils will get larger, even the pupil in the normal eye.
- It doesn't matter whether you start with the eye you think has the greater problem or the healthier eye: as long as the light is switched from one eye to the other and back again the signs should become apparent.

Sometimes the RAPD is obvious, as the pupil in the (most) affected eye very obviously gets larger when that eye is illuminated. But the signs can be more subtle (see Table 1).

## Specific situations

### Hippus

Normal pupils, particularly those of young people, sometimes show slight fluctuation in size (of less than 1 mm) even when the light shining into the eye is constant. This is called hippus and it can make eliciting a RAPD more difficult.

### Non-reactive pupils

**Figure 4:**
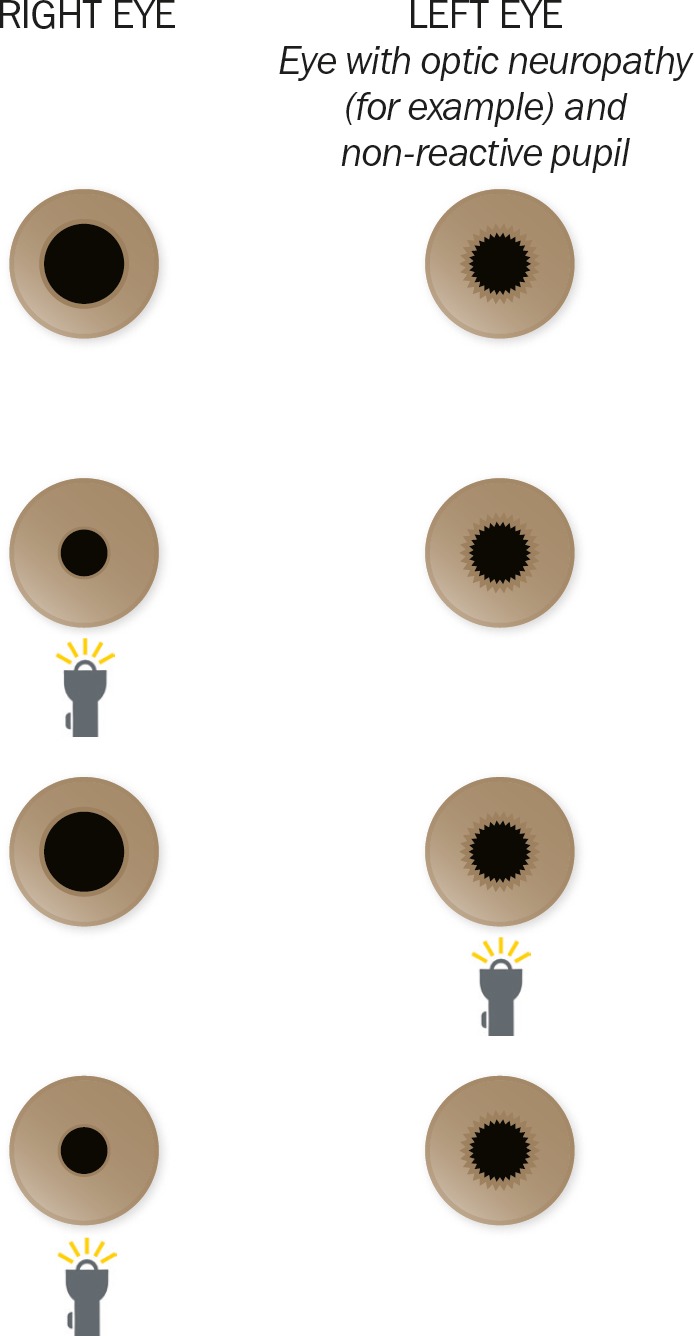
**Swinging-light test: left RAPD + non-reactive left pupil** Illumination of the relatively normal right eye causes only right pupil constriction. When the light is moved to the abnormal left eye (e.g. fixed pupil and optic neuropathy), the right pupil dilates (constricts less). Returning the light to the right eye results in constriction of the right pupil again. In this situation it is only necessary to observe the eye with the reactive pupil in order to identify an RAPD.

A RAPD can still be detected even if one pupil cannot change size (i.e. it is fixed), because of trauma, posterior synechiae or because dilating or constricting eye drops have been used (Figure 4). Having established that the pupil of one eye does not change size, regardless of which eye has the light shone into it, concentrate on the eye where the pupil is reactive. Note what happens to the reacting pupil when the light is shone into each eye in turn. Figure 4 shows what happens when the eye with the afferent pathway defect is also the eye with the fixed pupil. If the (more) normal eye is the one with the fixed pupil then, as the light moves from this eye to the other eye, the reacting pupil will dilate.

### Asymmetric refractive errors and/or amblyopia

These occur when the vision is poor but the eye itself is normal, and are not associated with a RAPD.

### Maculopathy

Unless very severe, this not usually associated with a RAPD and in eyes where the macular damage is sufficient to result in an RAPD, the grade is rarely more than 1–2 + (Table 1). Extensive retinal damage, major retinal vascular occlusion, or retinal detachment, by contrast, can lead to a high-grade RAPD.

**Table 1:** The grading of a RAPD in the swinging light test

**Amaurotic**	This is seen when one eye has no perception of light. The pupil of this eye only constricts when light is shone into the other eye. When the light is shone back into the eye with no perception of light the pupil rapidly enlarges against the light.
**3–4+**	The pupil enlarges as soon as the light is swung from the normal eye into the abnormal eye.
**1–2+**	The pupil enlarges, but only after a short delay, after the light is swung from the normal eye into the abnormal eye.
**Subtle/ trace**	Sometimes the pupils of both eyes can enlarge in the short time interval between shining the light in the normal eye and the abnormal eye. If this happens, the pupil of the abnormal eye may constrict a little bit before enlarging.

### Causes of RAPDs

Common causes of unilateral optic nerve disorders that can be associated with a RAPD include ischaemic optic neuropathy, optic neuritis, optic nerve compression (orbital tumours or dysthyroid eye disease), trauma, and asymmetric glaucoma. Less common suh causes include infective, infiltrative, carcinomatous, or radiation optic neuropathy. A RAPD is an extremely important localising clinical sign that can be detected by a simple, quick, non-invasive clinical test, provided that the test is performed carefully and correctly.
